# Predicting serious adverse events or a safety net – Rethinking the role of early warning scores

**DOI:** 10.1016/j.resplu.2023.100534

**Published:** 2023-12-30

**Authors:** Filip Haegdorens

**Affiliations:** Workforce Management and Outcome Research in Care (WORC) Group, Centre for Research and Innovation in Care, University of Antwerp (Belgium), Campus Drie Eiken – D.R.333, Universiteitsplein 1, BE-2610 Wilrijk, Belgium

To the editor,

The implementation of the National Early Warning Score (NEWS) in clinical practice has been the subject of much debate among researchers and clinicians worldwide. However, I want to argue that it is quite clear that an instrument such as the NEWS, that translates a combination of deviating vital signs into a single aggregated score, is a highly valuable tool for guiding healthcare providers in their patient care when applied thoughtfully.

I believe that researchers and clinicians have sometimes unrealistic expectations regarding Early Warning Scores. This is evident in a paper published in this journal by Thorén et al. who investigated the predictive power of the NEWS among patients assessed by a Rapid response team in a prospective multicentre trial across 26 Swedish hospitals.^1^ The authors concluded that the NEWS' performance fell short of the requirements for use as a risk stratification tool in patients assessed by a Rapid Response Team. Conversely, the NEWS performed much better in other studies including patients admitted to general wards to predict serious adverse events.^2^, ^3^

First, tools, such as the NEWS, were not originally developed as a risk stratification tool for patients already receiving specialized critical care. Instead, the objective was to provide an instrument that creates a ‘safety-net’ by detecting deviating vital signs in patients at risk for deterioration. Subsequently, rapid response teams can react to deviating scores and provide early critical care or initiate end-of-life care in the general ward aiming to avoid unnecessary and potentially harmful events such as unexpected intensive care unit admissions. Considering that one of the objectives is actually to reduce intensive care unit admissions, it is understandable that the NEWS's ability to accurately precisely predict this outcome in this specific population would be limited.

Second, I advocate for employing Early Warning Scores as a “ruling-out” tool rather than a “ruling-in” tool. As discussed before, a NEWS ≥ 5 generates a significant number of false positives and could exacerbate workload if implemented inadequately in low resource settings.^4^ However, if a patient has a NEWS < 5, we may confidently assume that in the next 24 hours this patient is less likely to experience a serious event. Ultimately, the decision to initiate a rapid response call should be made by the bedside nurse, physician, patient, or family members.^5^ NEWS is a valuable tool that, when coupled with clinical expertise and patient preferences, can aid healthcare providers in making substantiated decisions. One of the great challenges ahead is the implementation of automated tools that alert healthcare workers when scores are high without consulting the attending nurse. In my view, this approach would merely exacerbate the existing workload burden.

## Declaration of competing interest

The authors declare that they have no known competing financial interests or personal relationships that could have appeared to influence the work reported in this paper.
